# Facial EMG Correlates of Subjective Hedonic Responses During Food Consumption

**DOI:** 10.3390/nu12041174

**Published:** 2020-04-22

**Authors:** Wataru Sato, Kazusa Minemoto, Akira Ikegami, Makoto Nakauma, Takahiro Funami, Tohru Fushiki

**Affiliations:** 1 Kokoro Research Center, Kyoto University, 46 Shimoadachi, Sakyo, Kyoto 606-8501, Japan; minemoto.kazusa.6w@kyoto-u.ac.jp; 2 San-Ei Gen F. F. I., Inc., 1-1-11 Sanwa-cho, Toyonaka, Osaka 561-8588, Japan; akira-ikegami@saneigenffi.co.jp (A.I.); m-nakauma@saneigenffi.co.jp (M.N.); tfunami@saneigenffi.co.jp (T.F.); 3 Faculty of Agriculture, Ryukoku University, 1-5 Seta Oe-Cho Koya, Ohtsu, Shiga 520-2194, Japan; tfushiki@agr.ryukoku.ac.jp

**Keywords:** facial electromyography (EMG), food, liking, wanting, valence

## Abstract

An exploration of physiological correlates of subjective hedonic responses while eating food has practical and theoretical significance. Previous psychophysiological studies have suggested that some physiological measures, including facial electromyography (EMG), may correspond to hedonic responses while viewing food images or drinking liquids. However, whether consuming solid food could produce such subjective–physiological concordance remains untested. To investigate this issue, we assessed participants’ subjective ratings of liking, wanting, valence, and arousal while they consumed gel-type food stimuli of various flavors and textures. We additionally measured their physiological signals, including facial EMG from the corrugator supercilii. The results showed that liking, wanting, and valence ratings were negatively correlated with corrugator supercilii EMG activity. Only the liking rating maintained a negative association with corrugator supercilii activity when the other ratings were partialed out. These data suggest that the subjective hedonic experience, specifically the liking state, during food consumption can be objectively assessed using facial EMG signals and may be influenced by such somatic signals.

## 1. Introduction

The physiological correlates of subjective hedonic experiences (e.g., liking and wanting) during food consumption is important practically and theoretically. In terms of practicality, physiological measures are expected to provide objective, unbiased measures of hedonic responses. Although food product companies mainly rely on subjective hedonic ratings to develop new foods, subjective ratings can have biases, such as demand characteristics [1]. Theoretically, physiological activity may be an important element producing hedonic experiences while eating. Although this idea appears not to be frequently discussed in food preference studies, James [2] and several subsequent researchers [3,4] have proposed that physiological activation constitutes the essence of the subjective experience of emotion.

Some clues suggest the possibility that facial electromyography (EMG) activity may be associated with hedonic responses during food consumption. Many psychophysiological studies have revealed that facial EMG activity while participants observe emotional images reflects subjective emotional valence, which ranges from positive to negative and represents the qualitative component of emotion in a dimensional perspective [5,6]. Specifically, EMG activity recorded from the corrugator supercilii and zygomatic major muscles, which are related to brow lowering and lip corner pulling actions, respectively [7], are negatively and positively associated with valence ratings. For example, a previous study assessed subjective ratings of valence and arousal and measured some physiological signals, including facial EMG, while participants observed 21 emotional-scene images, including an image of food [8]. As a result, the subjective valence ratings showed linear negative and positive associations with corrugator supercilii and zygomatic major EMG activities, respectively. Also, one study measured facial EMG from certain facial muscles while participants consumed liquid stimuli (e.g., a sucrose solution) and showed a negative association between valence ratings and corrugator supercilii EMG activity [9]. Other studies suggested such an association between subjective hedonic reactions and facial EMG activity during consumption of liquid stimuli by showing EMG activity in these muscles [10,11] and by showing a negative association between palatability ratings and EMG activity in a different muscle (i.e., the levator labii) [12]. Other studies analyzed video data of facial expressions during the consumption of liquid stimuli and reported that facial expressions change depending on taste [13,14,15,16,17]. These data suggest that subjective hedonic experiences in relation to taste could be associated with facial EMG activity.

The concordance between subjective hedonic experiences and facial EMG activity while eating food is theoretically supported by evidence from two different fields of study. First, studies in human infants and animals have provided ample evidence that hedonic taste stimuli induce innate facial expressions [18]. Neuroscience studies in animals have identified the limbic and brainstem regions involved in the production of facial expressions to taste stimuli [19]. Second, several experimental psychological studies showed that the production of emotional facial expressions elicits corresponding subjective emotional experiences [20]. Collectively, these findings suggest that facial expressions evoked by food stimuli, which can be reflected in facial EMG signals, are causally related to subjective hedonic experience of eating.

However, it remains unknown whether facial EMG activity could be associated with hedonic responses during the consumption of solid food because no study has investigated this issue. Although a recent study analyzed video data and reported that facial expressions changed while eating solid food (i.e., smoked ham), the concordance between subjective hedonic ratings and facial expressions was not tested [21]. In addition, because a previous EMG study that tested the effect of liquid taste stimuli [9] analyzed the data only at the group level (i.e., calculated correlations between averaged/aggregated data), the data needed to be analyzed at the individual level to reveal intraindividual processes [22,23,24,25]. Based on the aforementioned data using emotional images and liquid stimuli, we hypothesized that subjective hedonic ratings would show individual-level associations with facial EMG activity during food consumption.

To investigate this hypothesis, we assessed subjective hedonic ratings and measured physiological signals during the consumption of solid food. As stimuli, we used bite-sized gel-type food materials with various flavors and textures. We manipulated flavors and textures because several studies have shown that food preferences are determined by a combination of these qualities [26]. Moreover, it is known that textures of foods affect flavor release; in general, the higher the fracture force of a foods, the lower the flavor release [27,28,29,30]. We requested participants to masticate, taste without mastication, and rate the stimuli according to the visual instructions. We assessed liking and wanting as subjective ratings, which have been used in many studies that tested hedonic responses to food [31] as well as valence and arousal, which have been assessed in many emotional studies [5,6]. Several previous studies found that the liking and wanting ratings generally were positively correlated during visual and oral food processing tasks [32], although some studies found a dissociation between these ratings under certain conditions [33,34,35]. We measured facial EMG from the corrugator supercilii and zygomatic major muscles as the physiological measures of interest. In addition, we exploratorily measured EMG of the masseter and suprahyoid muscles, which are associated with mastication of solid food [36,37]. We also exploratorily measured skin potential level (SPL) and heart rate (HR), which are assessments of the autonomic nervous system and generally reflect emotional arousal [5,6,38], although previous studies did not show consistent hedonic reactions to liquid stimuli for these parameters [13,15,39]. We calculated intraindividual correlation coefficients between the subjective ratings and physiological activity during the tasting periods and evaluated the coefficients at second-level group analyses. We expected that liking, wanting, and valence ratings would have negative and positive associations with corrugator supercilii and zygomatic major EMG activities, respectively.

## 2. Materials and Methods

### 2.1. Participants

Twenty-four Japanese volunteers (10 females; mean ± SD age, 22.8 ± 5.8 years) participated in this study. The required sample size was determined based on a preliminary experiment with a different sample (*n* = 12) that tested the concordance between hedonic ratings and facial EMG responses to visual food images and an a priori power analysis using G*Power software ver. 3.1.9.2 [40]. The effect size was estimated from the results, and an *α* level of 0.05 with power (1 − *β*) of 0.80 was assumed. The result of the power analysis showed that more than 22 participants were needed. All participants were blinded to the research objectives and had fasted for more than 3 h before the experiments. Their hunger levels were assessed before the experiments using a 5-point scale ranging from 1 (hungry) to 5 (full), and the results indicated that none of them were full (<5) and that the majority of them were relatively hungry (mean ± SD, 2.8 ± 0.7). None of the participants were obese (body mass index, <30, mean ± SD, 20.6 ± 2.1 kg/m^2^). After a detailed explanation of the experimental procedure, all participants provided informed consent. Our study was approved by the Ethics Committee of the Unit for Advanced Studies of the Human Mind, Kyoto University (30-P-6) on September 21, 2018. This experiment was conducted following institutional ethical provisions and the Declaration of Helsinki.

### 2.2. Stimuli

**Figure 1:**
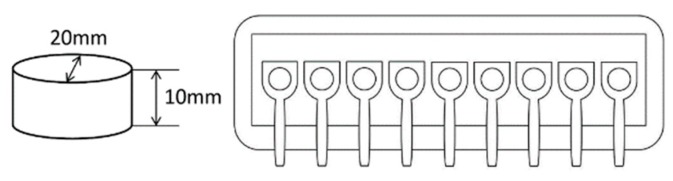
Schematic illustrations of the stimuli (left) and experimental setups (right): The bite-sized gel-type food stimuli were placed on disposable plastic spoons in a row.

We used 18 bite-sized gel-type solid food materials with six flavor types and three texture types (Figure 1). All stimuli contained 10% sucrose; 0.06% anhydrous citric acid; 0.03% trisodium citrate; 0.1% calcium lactate; and 0.25%, 0.35%, or 0.5% low-acetylated gellan gum (Kelcogel, provided by San-Ei Gen F.F.I., Osaka, Japan). In addition, the stimuli contained six specific flavoring agents, two of each are generally evaluated as negative, neutral, and positive hedonic traits. The agents were 0.001% isovaleric acid (Tokyo Chemical Industry, Tokyo, Japan) and 0.02% butanol (Kishida Chemical, Osaka, Japan) for negative, no flavor and 0.006% linalool (Kanto Chemical, Tokyo, Japan) for neutral, and 0.02% vanillin (Tokyo Chemical Industry, Tokyo, Japan) and 0.02% limonene (Fujifilm Wako Pure Chemical, Osaka, Japan) for positive traits. All of these flavors are used in commercial food materials. The concentrations of the flavoring agents were determined in a series of preliminary experiments. The final preliminary experiment, which included six participants who did not take part in the subsequent experiment, found max–min ranges ≥4 in the liking ratings for all participants. It should be noted that flavor preferences generally show large individual differences in food preference studies [41]; we were interested in testing the physiological correlates of individuals’ specific hedonic experiences. A mixture of sucrose and gellan gum was added to deionized water at 90 °C in 500-mL glass beakers and stirred at 1300 rpm for 10 min at the same temperature. Then, the calcium lactate, anhydrous citric acid, and trisodium citrate were added to the solutions. The solutions were poured into plastic cups (65 mm in diameter and 25 mm in height) inside which cylindrical glass molds (20 mm in diameter and 10 mm in height) were already placed. The solutions in the cups were hermetically sealed, heated at 85 °C for 30 min, and refrigerated at 8 °C for 1 h. The size of the gels prepared was 20 mm in diameter and 10 mm in height. The differences in the texture types were based on the proportion of low-acetylated gellan gum. The fracture strain of all gels with different proportions of low-acetylated gellan gum was approximately equivalent at 50%, whereas the fracture forces of gels with 0.25%, 0.35%, and 0.5% gellan gum were approximately 10, 15, and 20 N, respectively. Fracture strain and forces were measured using TA-XT2i texture analyzer (Stable Micro Systems, Surrey, UK). Fracture force and fracture strain were determined by compressing the gels on a metal stage using a 75-mm diameter aluminum plate at a crosshead speed of 10 mm/s at 20 °C.

### 2.3. Procedure

Experiments were conducted individually in an electrically shielded soundproof room (Science Cabin, Takahashi Kensetsu, Tokyo, Japan). The instructions and response recording were controlled using Presentation software (Neurobehavioral Systems, Berkeley, CA, USA) on a Windows computer (HP Z200 SFF, Hewlett-Packard Japan, Tokyo, Japan).

Upon arrival, participants were told that the experiment was being conducted to test electric physiological activity during food eating. The nine gel-type food stimuli were placed on a dish on the desk in front of a computer monitor (Figure 1). The dish was replaced by another dish with nine different food stimuli at a break. The stimuli were set on 8-cm disposable plastic spoons, one by one, in a row to allow participants to eat them with one hand in a predefined order (from right to left). To avoid contaminating the flavored water, each stimulus was set with enough space around it and a piece of paper was laid under the materials. All stimuli were set up about 10 min before the experiment. After two practice trials, 18 trials were conducted and a break was taken after half of the trials. The order of stimuli was pseudo-random with no repetition of general hedonic traits. The intertrial interval was randomly changed between 20 and 30 s.

For each trial, a small white cross was presented on a black background on a computer monitor as a warning period signal for 3 s. Then, a large red cross was presented as a mouthing and masticating period signal for 5 s followed by a blue large cross as a tasting period signal for 5 s. Then, a response panel with four rating scales (liking, wanting, valence, and arousal) was presented until the responses were finished. The participants were instructed to (1) prepare to mouth the stimuli with the white cross; (2) to mouth the stimuli as soon as the red cross appeared and to masticate while the red cross was displayed; (3) to taste without masticating while the blue cross was displayed; and (4) to re-masticate, to swallow the stimuli, and to rate their hedonic experiences when they ate the stimuli by pressing the appropriate key when the response panel appeared. Four 9-point rating scales (liking, wanting, valence, and arousal) were presented simultaneously in this fixed order. Liking and wanting were rated using the labels of these terms and lines with numbers and wording of 1 (dislike) to 9 (like) and 1 (do not want to eat) to 9 (want to eat), respectively. Valence and arousal were rated using the labels of these terms and numbers with the images of self-assessment manikins [42].

### 2.4. Physiological Data Recording

**Figure 2:**
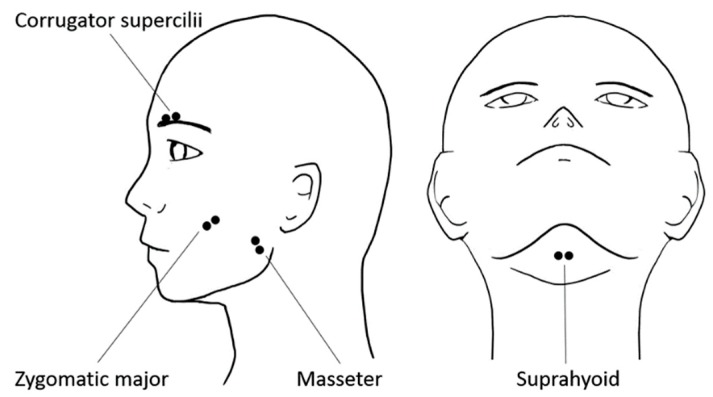
Schematic illustrations of electrode placement for electromyography recording of the corrugator supercilii, zygomatic major, masseter, and suprahyoid muscles.

Facial EMG data were recorded from the corrugator supercilii, zygomatic major, masseter, and suprahyoid muscles (Figure 2). Sets of pre-gelled, self-adhesive 0.7-cm diameter Ag/AgCl electrodes with 1.5-cm inter-electrode spacing (Prokidai, Sagara, Japan) were used. The electrodes were placed according to guidelines [43,44] and previous studies [36,37]. A ground electrode was placed on the forehead. The data were amplified, filtered online (bandpass: 20–400 Hz [45]), and sampled at 1000 Hz using an EMG-025 amplifier (Harada Electronic Industry, Sapporo, Japan) and the PowerLab 16/35 data acquisition system and LabChart Pro v8.0 software (ADInstruments, Dunedin, New Zealand). A video recording was unobtrusively made using a digital web camera (HD1080P, Logicool, Tokyo, Japan) to check for motion artifacts and facial positions.

SPL data were recorded from the palms using pre-gelled, self-adhesive 1.0-cm Ag/AgCl electrodes (Vitrode F; Nihonkoden, Tokyo, Japan). The probe and reference electrodes were placed on the hypothenar site of the left palm and the left forearm, respectively, of the participants according to guidelines [38]. The data were amplified using SPN-01 amplifier (Skinos, Ueda, Japan) and recorded with the same data acquisition system and recording software as the aforementioned EMG recording, except no online filter was used. SPL is a measure of eccrine sweat gland activity, which is controlled by the sympathetic branch of the autonomic nervous system, specifically, its tonic component [38]. Basal SPL is typically in the negative range, and relaxation decreases this negativity [46]. We assessed the tonic component as in a previous study [13] because our experiment involved multiple behavioral responses (biting and chewing) before the recording period, which could affect the phasic components, and because we were interested in continuous states for a specific period of time (5 s). However, because the amplifier produced the phasic skin potential response (SPR) output, we also assessed this component (see below).

HR was recorded using a photoplethysmograph (MLT1020FC, ADInstruments, Dunedin, New Zealand) positioned on the last phalange of the left second finger. The data were recorded using the same data acquisition system and recording software with the aforementioned EMG recording, except no online filter was used. The software automatically calculated beats per minute. HR is controlled by the sympathetic and parasympathetic branches of the autonomic nervous system [6].

### 2.5. Data Analysis

#### 2.5.1. Preprocessing

EMG data analyses were performed using Psychophysiological Analysis Software 3.3 (Computational Neuroscience Laboratory of the Salk Institute) and in-house programs implemented in MATLAB 2018 (MathWorks, Natick, MA, USA). The data were sampled during the pre-stimulus baseline period for 0.5 s immediately before the stimulus presentation (the fixation was presented without food consumption) and the tasting period (food tasting without mastication) for 5 s during each trial. One of the authors blindly checked the video data and confirmed that the participants did not produce large motion artifacts. The data for each trial were rectified, baseline-corrected to the average value over the pre-stimulus period, and averaged. The values for each stimulus were then standardized within each individual. Data beyond |3| (i.e., >3 |SD|) were removed to eliminate the effect of outliers.

Identical analyses were conducted for the SPL and HR data as for the EMG analysis except that the data were not rectified. Averaging is the recommended statistical approach for the assessment of SPL and HR data [47,48].

#### 2.5.2. Statistical Analysis

To evaluate the individual-level concordance between subjective hedonic ratings and physiological activity, we calculated Pearson’s product–moment correlation coefficients between subjective ratings and mean physiological activity during the tasting period without mastication in each participant. We predicted and analyzed the relationships between liking/wanting/valence and corrugator supercilii/zygomatic major EMG. We also analyzed other relationships in the same manner for descriptive purposes. The correlation coefficients were normalized using Fisher’s *r*-to-*z* transformation and entered into one-sample *t*-tests (two-tailed) to test for significant differences from zero, as in previous studies that tested subjective–physiological emotional concordance with non-food stimuli (e.g., Reference [49]). Such random-effect analyses via a two-stage procedure are used to evaluate the generalizability of individual-level statistical models and are widely used in the neuroimaging community [50]. Because one participant showed a constant value for the liking ratings across all trials, the data of this participant were removed from the liking data analyses. To illustrate visually the relationships between subjective hedonic ratings and physiological activity at the group-level, we depicted the concordance between group-averaged subjective and physiological data. The ratings showing significant subjective physiological correlations were calculated as intraindividual partial correlations to control for the effects of the other subjective ratings and were assessed using one-sample *t*-tests as in the previous analyses. The results were considered significant at *p* < 0.05.

We performed a preliminary analysis on the SPR data. We calculated the absolute maximum SPR values during the tasting period; the correlation analysis revealed no significant associations of SPR with any subjective ratings (*p* > 0.10). Therefore, we only report the SPL findings.

## 3. Results

The mean ± SD subjective ratings and physiological activity in response to each stimulus are shown in Tables S1 and S2 and Figures S1 and S2. As we expected, based on previous findings [41] and those of our preliminary experiment, the subjective ratings revealed large individual differences across flavors and textures. For example, although isovaleric acid combined with the 15 N texture was generally the least liked sample (mean ± SD, 4.25 ± 1.91), one participant rated it as 8, which was his second highest score. Whereas vanillin with the 10 N texture was generally the most liked sample (mean ± SD, 6.04 ± 1.60), one participant rated it as 2, his second lowest rating.

**Figure 3:**
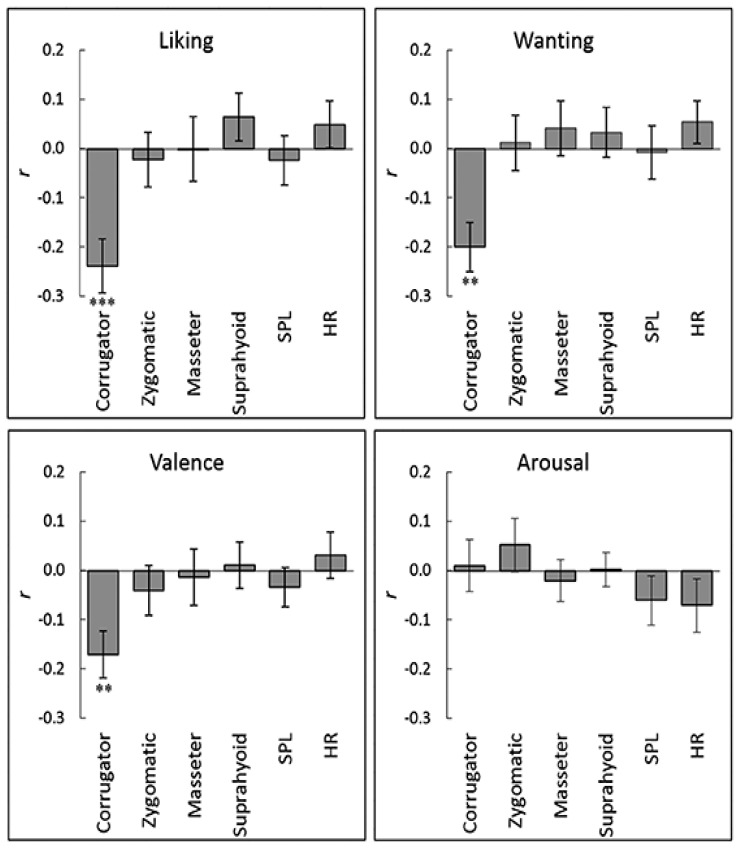
Mean (with standard error) intraindividual correlation coefficients between subjective ratings and physiological responses across stimuli: Corrugator = corrugator supercilii; Zygomatic = zygomatic major; SPL = skin potential level; HR = heart rate. ***, *p* < 0.001; **, *p* < 0.005.

**Figure 4:**
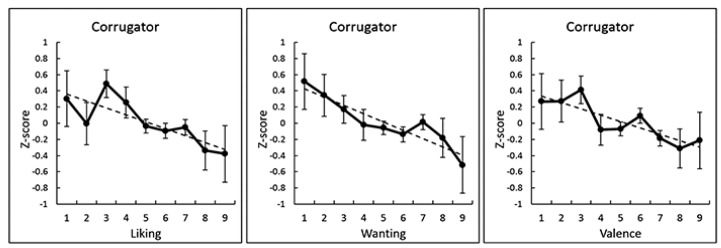
Group-mean (with standard error) values and regression lines of subjective ratings (liking, wanting, and valence) and corrugator supercilii electromyography activity (standardized within each individual).

The correlation coefficients between subjective hedonic ratings and mean physiological responses during the tasting period were calculated for each participant to analyze statistically intraindividual subjective–physiological concordance during food consumption (Figure 3). Then, one-sample *t*-tests were conducted to identify a significant difference from zero in the correlation coefficients after the Fisher’s *r*-to-*z* transformation. The results showed that the liking, wanting, and valence ratings had significant negative associations with corrugator supercilii EMG activity (*t* > 3.56, *p* < 0.005; Table 1). Figure 4 illustrates these relationships as group-averaged values. No other significant associations between subjective ratings and physiological responses were detected (*p* > 0.10).

**Figure 5:**
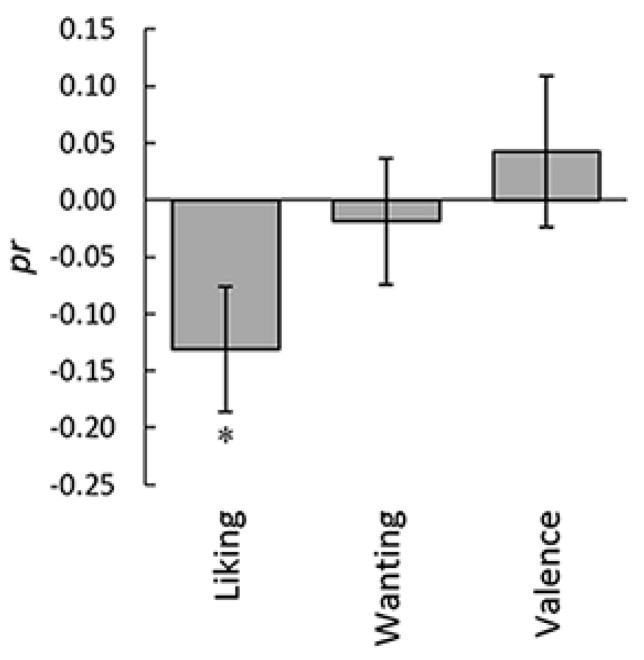
Mean (with standard error) intraindividual partial correlation coefficients between subjective ratings and corrugator supercilii electromyography activity, controlling for the other two ratings. * *p* < 0.05.

Because the three states that were significantly associated with corrugator supercilii EMG activity (i.e., liking, wanting, and valence) were themselves strongly positively correlated (e.g., liking–wanting, mean ± SE *r*, 0.81 ± 0.04, *p* < 0.001; Table S3), we investigated whether the associations with EMG activity remained significant after controlling for the effects of the other ratings. We calculated the intraindividual partial correlations for a single measure, partialling out the remaining two (Figure 5). One-sample *t*-tests performed after the Fisher’s *r*-to-*z* transformation revealed a significant negative partial correlation of corrugator supercilii EMG activity with the liking rating (*t*(22) = 2.45, *p* < 0.05) but not with the wanting and valence ratings (*t*(22) < 0.87, *p* > 0.10).

## 4. Discussion

Our results demonstrated that higher ratings of liking, wanting, and valence were associated with lower EMG activity in the corrugator supercilii muscle during consumption of gel-type food stimuli. These results are consistent with the findings of several previous studies showing negative associations between subjective valence/hedonic ratings and corrugator supercilii muscle EMG activity using emotional images [5,6] and liquid stimuli [9]. However, no study has investigated this issue using solid food materials. Furthermore, a study testing the effect of taste did not evaluate individual-level correlations [9]. To the best of our knowledge, this study provides the first evidence that subjective hedonic experiences during consumption of solid food show individual-level negative associations with facial EMG activity at the corrugator supercilii muscle.

The partial correlations revealed that, when the other ratings were controlled for, the liking rating was significantly associated with corrugator supercilii EMG activity, whereas the wanting and valence ratings were not. Interestingly, these findings are consistent with previous neuroscience studies in animals showing that the brain regions and neurotransmitters involved in producing facial expressions (liking reactions) and motivating eating behaviors (wanting process) can be dissociated [19]. Although liking and wanting ratings typically overlap in humans [32], some studies have revealed differing patterns; for example, when the ratings for food were compared before and after a lunch eaten to satiety, the wanting rating decreased more than the liking rating [35]. Our finding that liking was more closely associated with facial muscle reactions while eating food than wanting provides new supportive evidence for the dissociation between the liking and wanting experiences in humans.

In contrast to our expectation, our results did not reveal significant positive associations between subjective hedonic ratings and zygomatic major EMG activity. These results suggest the possibility that the hedonic processing of food does not activate the zygomatic major muscles. However, it might be that our experimental procedures, such as tasting without mastication instructions, suppressed EMG activity in the zygomatic major muscle associated with a hedonic experience. Our SPL and HR results did not show significant associations with subjective ratings, which is consistent with previous findings [15]. However, the directions of the nonsignificant associations between arousal ratings and these measures were theoretically valid. Specifically, higher arousal induced negative deflection in SPL, which reflects the activity of the sympathetic nervous system [38] and a decrease in HR, which are generally shown while observing emotionally arousing images [5,6]. Hence, it may be possible that food stimuli that can induce more arousing hedonic experiences than our stimuli may reveal associations between subjective arousal and activity in these measures. Collectively, further studies are needed to make a conclusion regarding the associations between subjective hedonic experiences and zygomatic major EMG and autonomic nervous system measures during consumption of food.

Our results, showing clear facial EMG correlates of hedonic subjective experiences during food consumption, have practical implications. Because hedonic reactions are important for eating behaviors [51], food product companies try to assess hedonic reactions to their new products. However, companies mainly rely on subjective ratings, which can have biases, such as demand characteristics [1]. Our data suggest that facial EMG provides objective support for subjective hedonic ratings. Furthermore, EMG recordings have advantages compared with subjective ratings, such as detecting subtle emotional reactions that cannot be subjectively experienced or cannot be aware of triggering signals [52,53], which can occur with food stimuli [54,55,56,57]. Furthermore, using facial EMG as an estimate of subjective hedonic experiences may be useful when participants are unable to report their hedonic experiences, such as infants and individuals with dementia. However, it should be noted that facial EMG has several drawbacks compared with subjective ratings. For example, recording facial EMG requires that electrodes be placed on participants’ faces, which can be disturbing [58]. Video recordings of facial expressions may be preferable for assessing facial reactions during eating, although video recordings are less sensitive than EMG [58]. Therefore, under conditions that require monitoring of unbiased or subtle hedonic states during eating, facial EMG may complement subjective ratings or other assessment methods and may facilitate the detection of hedonic states evoked by food.

Our results also have theoretical implications. The concordance between subjective and physiological emotional responses has long been of interest to the psychology of emotion, ever since James [2] proposed that physiological signals are the substance of subjective emotional experiences, though there remain debates [3,4]. In contrast, studies on the psychological and neural mechanisms of hedonic responses to food have mainly focused on evaluating the stimulus and motivation elicitation in the brain [19] and has paid less attention to the influence of somatic responses. Our data suggest the possibility that facial muscle contraction and its interoception, associated with activity in the brainstem, hypothalamus, and somatosensory areas [59,60,61], may play an important role in the production of subjective hedonic experiences during eating.

Our study has several limitations. First, our instructions requested participants to taste food without chewing. Although we used this manipulation to remove artifacts in the EMG related to mastication, it induced an unnatural way to consume food. Hence, it remains unproven whether corrugator supercilii EMG activity could reflect subjective hedonic experiences during natural eating. Also, as described above, the instruction not to chew the food but to hold it in the mouth may have produced less evident zygomatic major EMG activity. Moreover, as the participants were instructed only not to chew, their tongue movement may have affected the EMG signal. Future studies are needed to investigate the physiological correlates of subjective hedonic experiences without restrictions or with more rigorous control of motion artifacts during the consumption of solid food. This may be accomplished by measuring the physiological responses during reflection periods just after food is consumed. Second, our stimuli were restricted to gel-type food materials. We used these to control low-level perceptual features across conditions as the first step in the investigation of subjective and physiological associations with solid food. Furthermore, we mainly differentiated the valence of flavors, which may, in part, explain the lack of association between subjective arousal and physiological measurements. Future studies are needed to test different food items, including those common in daily life and food with various arousal properties. Third, our small sample of young people may limit the generalizability of our findings. Our findings need to be replicated in studies with larger samples that include a wider age range. Studies of older individuals may extend the application of our findings to real-life situations, such as elder care. Finally, the magnitude of the correlation coefficients between subjective hedonic experiences and corrugator supercilii EMG activity were weak (<0.20) to moderate (0.20–0.30) according to empirical guidelines [62]. An increase in sensitivity would be needed for practical application of our findings. Artifact rejection using statistical methods, such as independent component analysis [63], may remove artifacts related to mouth movements and may increase the signal-to-noise ratio. Nonlinear analysis using machine learning [64] may more sensitively detect associations between subjective hedonic experiences and facial EMG activity while eating food.

## 5. Conclusions

Our results demonstrated that the subjective ratings of liking, wanting, and valence were negatively correlated with facial EMG activity recorded from the corrugator supercilii during the tasting of gel-type solid food stimuli. The liking rating was the only one that showed a negative association with corrugator supercilii activity when the other ratings were partialed out. Our findings suggest that subjective hedonic experience, specifically the liking component, during food consumption can be objectively assessed using facial EMG signals and may be influenced by the feedback of such somatic signals.

## Figures and Tables

**Table 1:** Results of one-sample *t*-test (two-tailed) for intraindividual correlation coefficients between subjective ratings and physiological responses.

Subjective	Statistic	Physiological					
		Corrugator	Zygomatic	Masseter	Suprahyoid	SPL	HR
Liking	*t*	**4.31**	0.53	0.14	1.29	1.09	0.60
	*p*	**0.000**	0.602	0.893	0.211	0.289	0.552
Wanting	*t*	**3.98**	0.08	0.42	0.72	1.22	0.29
	*p*	**0.001**	0.936	0.680	0.481	0.234	0.775
Valence	*t*	**3.56**	0.91	0.31	0.33	0.70	0.88
	*p*	**0.002**	0.373	0.760	0.744	0.492	0.389
Arousal	*t*	0.27	1.52	0.47	0.11	1.23	1.22
	*p*	0.788	0.143	0.646	0.916	0.231	0.235

Degrees of freedom are 23 for all except liking (22). Significant results (*p* < 0.05) are in bold. Corrugator = corrugator supercilii; Zygomatic = zygomatic major; SPL = skin potential level; HR = heart rate.

## Supplementary Materials

The following are available online at https://www.mdpi.com/2072-6643/12/4/1174/s1, Table S1: Mean (with SD) subjective ratings, Table S2: Mean (with SD) physiological activity, Table S3: Mean (with SE) correlation coefficients among the subjective ratings, Figure S1: Mean (with SD) subjective ratings, Figure S2: Mean (with SD) physiological activity.
