# Supplementary material for: Facial EMG Correlates of Subjective Hedonic Responses During Food Consumption
Source: Nutrients. 2020 Apr 22;12(4):1174. doi: 10.3390/nu12041174 (PMC7230625; doi:10.3390/nu12041174)
Supplement: Supplementary file 1 [file nutrients-12-01174-s001.zip › TableS1S2/SuppTable1_200318.doc]

Table S1. Mean (with *SD*) subjective ratings.

| Stimulus | |  | Measure | | | |
| --- | --- | --- | --- | --- | --- | --- |
| Flavor | Texture (N) |  | Liking | Wanting | Valence | Arousal |
| Isovaleric acid | 10 |  | 4.38 | 3.54 | 3.83 | 5.00 |
|  |  |  | (1.68) | (1.88) | (1.85) | (1.97) |
|  | 15 |  | 4.25 | 3.58 | 4.25 | 5.42 |
|  |  |  | (1.91) | (2.16) | (2.25) | (1.86) |
|  | 20 |  | 4.63 | 3.92 | 4.13 | 4.54 |
|  |  |  | (1.31) | (1.55) | (1.42) | (1.61) |
| Butanol | 10 |  | 5.88 | 5.54 | 5.54 | 5.33 |
|  |  |  | (1.65) | (1.66) | (1.47) | (1.49) |
|  | 15 |  | 5.75 | 5.71 | 5.58 | 5.17 |
|  |  |  | (1.42) | (1.73) | (1.34) | (1.65) |
|  | 20 |  | 5.54 | 5.29 | 5.25 | 4.96 |
|  |  |  | (1.69) | (1.89) | (1.56) | (1.75) |
| No | 10 |  | 5.17 | 4.96 | 4.96 | 5.58 |
|  |  |  | (1.63) | (1.98) | (1.85) | (1.52) |
|  | 15 |  | 5.00 | 4.38 | 4.71 | 5.13 |
|  |  |  | (1.84) | (2.06) | (2.01) | (1.82) |
|  | 20 |  | 5.00 | 4.46 | 4.71 | 4.92 |
|  |  |  | (1.10) | (1.47) | (1.45) | (1.13) |
| Linalool | 10 |  | 5.67 | 5.25 | 5.50 | 4.67 |
|  |  |  | (1.23) | (1.87) | (1.28) | (1.60) |
|  | 15 |  | 5.21 | 4.96 | 5.17 | 4.08 |
|  |  |  | (1.14) | (1.70) | (1.40) | (1.79) |
|  | 20 |  | 5.13 | 4.92 | 5.04 | 4.38 |
|  |  |  | (1.51) | (1.58) | (1.45) | (1.68) |
| Vanillin | 10 |  | 6.04 | 5.58 | 5.71 | 4.88 |
|  |  |  | (1.60) | (1.97) | (1.33) | (1.82) |
|  | 15 |  | 5.54 | 5.17 | 5.58 | 5.04 |
|  |  |  | (1.74) | (1.90) | (1.81) | (1.57) |
|  | 20 |  | 5.71 | 5.17 | 5.58 | 5.29 |
|  |  |  | (1.42) | (1.73) | (1.31) | (1.75) |
| Limonene | 10 |  | 5.96 | 5.50 | 6.00 | 5.29 |
|  |  |  | (1.23) | (1.84) | (1.25) | (1.73) |
|  | 15 |  | 5.42 | 5.21 | 5.50 | 5.08 |
|  |  |  | (1.50) | (1.86) | (1.50) | (1.17) |
|  | 20 |  | 5.42 | 4.88 | 5.25 | 4.83 |
|  |  |  | (1.47) | (1.72) | (1.35) | (1.49) |
