# Supplementary material for: Facial EMG Correlates of Subjective Hedonic Responses During Food Consumption
Source: Nutrients. 2020 Apr 22;12(4):1174. doi: 10.3390/nu12041174 (PMC7230625; doi:10.3390/nu12041174)
Supplement: Supplementary file 1 [file nutrients-12-01174-s001.zip › TableS1S2/SuppTable2_200318.doc]

Table S2. Mean (with *SD*) physiological activity.

| Stimulus | |  | Measure | | | | | |
| --- | --- | --- | --- | --- | --- | --- | --- | --- |
| Flavor | Texture (N) |  | Corrugator | Zygomatic | Masseter | Suprahyoid | SPL | HR |
| Isovaleric acid | 10 |  | 0.19 | 0.13 | –0.43 | –0.19 | 0.11 | 0.08 |
|  |  |  | (0.74) | (1.16) | (1.16) | (0.81) | (1.09) | (1.06) |
|  | 15 |  | 0.46 | 0.20 | –0.21 | –0.18 | –0.12 | 0.09 |
|  |  |  | (1.22) | (1.16) | (0.90) | (0.88) | (0.80) | (0.73) |
|  | 20 |  | 0.25 | –0.11 | 0.06 | –0.06 | 0.02 | 0.09 |
|  |  |  | (1.11) | (1.12) | (0.96) | (0.64) | (1.11) | (0.61) |
| Butanol | 10 |  | –0.07 | –0.16 | –0.22 | 0.09 | –0.11 | 0.06 |
|  |  |  | (0.90) | (0.81) | (0.61) | (1.22) | (1.11) | (0.79) |
|  | 15 |  | –0.20 | 0.29 | 0.10 | 0.12 | –0.07 | –0.21 |
|  |  |  | (1.00) | (1.04) | (1.04) | (0.75) | (1.25) | (1.07) |
|  | 20 |  | –0.07 | –0.22 | 0.03 | –0.26 | 0.05 | 0.20 |
|  |  |  | (1.24) | (0.98) | (0.77) | (0.99) | (0.76) | (0.95) |
| No | 10 |  | –0.28 | 0.13 | –0.06 | –0.23 | 0.05 | –0.23 |
|  |  |  | (0.97) | (0.71) | (0.94) | (0.88) | (0.81) | (1.09) |
|  | 15 |  | –0.06 | –0.44 | –0.20 | 0.07 | 0.13 | 0.11 |
|  |  |  | (0.99) | (1.02) | (0.65) | (0.84) | (0.95) | (0.66) |
|  | 20 |  | 0.22 | –0.01 | 0.11 | –0.11 | –0.21 | –0.05 |
|  |  |  | (1.02) | (0.93) | (0.98) | (1.02) | (1.00) | (1.18) |
| Linalool | 10 |  | –0.30 | –0.26 | –0.06 | 0.24 | –0.25 | 0.02 |
|  |  |  | (0.66) | (0.72) | (0.71) | (0.65) | (0.75) | (0.85) |
|  | 15 |  | –0.10 | –0.24 | 0.05 | 0.04 | –0.07 | 0.05 |
|  |  |  | (1.01) | (0.84) | (1.20) | (1.05) | (0.82) | (0.78) |
|  | 20 |  | 0.29 | 0.37 | 0.38 | –0.20 | 0.05 | –0.23 |
|  |  |  | (0.91) | (1.18) | (0.64) | (1.14) | (1.00) | (1.03) |
| Vanillin | 10 |  | –0.15 | –0.23 | 0.02 | –0.06 | –0.05 | –0.22 |
|  |  |  | (0.64) | (0.90) | (1.06) | (0.67) | (1.06) | (1.31) |
|  | 15 |  | –0.17 | 0.04 | –0.25 | 0.15 | 0.30 | 0.27 |
|  |  |  | (0.93) | (1.04) | (0.98) | (1.15) | (1.06) | (0.71) |
|  | 20 |  | –0.30 | 0.10 | 0.25 | 0.15 | 0.02 | –0.07 |
|  |  |  | (1.11) | (0.87) | (1.11) | (1.36) | (0.91) | (1.18) |
| Limonene | 10 |  | –0.10 | 0.18 | 0.24 | 0.02 | 0.16 | 0.16 |
|  |  |  | (1.09) | (1.03) | (1.06) | (1.06) | (1.15) | (0.92) |
|  | 15 |  | 0.23 | 0.14 | –0.02 | 0.24 | –0.12 | –0.38 |
|  |  |  | (0.55) | (0.78) | (1.01) | (1.13) | (1.00) | (1.23) |
|  | 20 |  | 0.12 | 0.08 | 0.20 | 0.16 | 0.05 | 0.22 |
|  |  |  | (0.89) | (0.80) | (1.25) | (0.90) | (0.80) | (0.93) |

All physiological data were standardized within each individual. Corrugator = Corrugator supercilii; Zygomatic = zygomatic major; SPL = skin potential level; HR = heart rate.
